# Exogenous PTHrP Repairs the Damaged Fracture Healing of PTHrP+/− Mice and Accelerates Fracture Healing of Wild Mice

**DOI:** 10.3390/ijms18020337

**Published:** 2017-02-06

**Authors:** Yinhe Wang, Xin Fang, Chun Wang, Congzhu Ding, Hua Lin, Anlong Liu, Lei Wang, Yang Cao

**Affiliations:** 1 Department of Orthopaedic Surgery, Nanjing Drum Tower Hospital, Affiliated Hospital of Nanjing University Medical School, Nanjing 210008, China; lh2116@126.com (H.L.); liuanlongglyy@hotmail.com (A.L.); 2 Unit of Biostatistics, Institute of Environmental Medicine, Karolinska Institutet, Stockholm 17177, Sweden; yang.cao@ki.se; 3 Department of Geriatrics, Nanjing Drum Tower Hospital, Affiliated Hospital of Nanjing University Medical School, Nanjing 210008, China; wangchunglyy@hotmail.com (C.W.); dingcongzhuglyy@hotmail.com (C.D.); 4 Department of Oral & Maxillofacial-Head & Neck Oncology, The Ninth People’s Hospital, Shanghai Jiao Tong University School of Medicine, Shanghai Key Laboratory of Stomatology, Shanghai 200011, China; wanglei@sh9hospital.org; 5 Clinical Epidemiology and Biostatistics, School of Medical Sciences, Örebro University, Örebro 70182, Sweden

**Keywords:** bone fracture healing, parathyroid hormone-related protein (PTHrP), PTHrP+/− mice, exogenous, endogenous, callus tissue

## Abstract

Bone fracture healing is a complicated physiological regenerative process initiated in response to injury and is similar to bone development. To demonstrate whether an exogenous supply of parathyroid hormone–related protein (PTHrP) helps in bone fracture healing, closed mid-diaphyseal femur fractures were created and stabilized with intramedullary pins in eight-week-old wild-type (WT) PTHrP+/+ and PTHrP+/− mice. After administering PTHrP for two weeks, callus tissue properties were analyzed at one, two, and four weeks post-fracture (PF) by various methods. Bone formation–related genes and protein expression levels were evaluated by real-time reverse transcriptase–polymerase chain reaction and Western blots. At two weeks PF, mineral density of callus, bony callus areas, mRNA levels of alkaline phosphatase (ALP), type I collagen, Runt-related transcription factor 2 (Runx-2), and protein levels of Runx-2 and insulin-like growth factor-1 decreased in PTHrP+/− mice compared with WT mice. At four weeks PF, total collagen-positive bony callus areas, osteoblast number, ALP-positive areas, and type I collagen-positive areas all decreased in PTHrP+/− mice. At both two and four weeks PF, tartrate-resistant acid phosphatase–positive osteoclast number and surface decreased a little in PTHrP+/− mice. The study indicates that exogenous PTHrP provided by subcutaneous injection could redress impaired bone fracture healing, leading to mutation of activated PTHrP by influencing callus areas, endochondral bone formation, osteoblastic bone formation, and bone turnover.

## 1. Introduction

Bone fracture healing is a complicated physiological regenerative process initiated in response to injury and is similar to bone development. In the initial phase of fracture repair, various growth factors produced by the injured tissues affect committed osteoprogenitor cells and undifferentiated mesenchymal cells at the site of injury, resulting in hard callus formation and an increase in the strength at the site of fractured bone [1]. Bone fracture healing has been recognized as a long-term process of ossification and remodeling subsequent to hematoma formation, inflammatory response, and cell proliferation and differentiation [2]. To achieve regeneration of a fully functional bone, many interrelated anatomical, biomechanical, and biochemical processes must occur in a well-coordinated fashion.

Despite present improvements in fracture management, delayed union and nonunion remnants are still incurable complications following surgical reduction and fixation of bone fractures. It was reported that 5%–10% of all fractures display impaired healing [3]. Currently, autogenous bone graft has been attested to be the gold standard for treating delayed fracture union, but it is accompanied with multiple complications [4,5]. In addition, nonsurgical methods, such as drug use, are also considered to facilitate osteogenesis and, hence, to revitalize the healing process when a delayed union occurs [6,7]. In recent years, many biological factors have been noted to promote bone fracture healing, and more factors such as vascular endothelial growth factor, bone morphogenetic proteins, macrophage colony-stimulating factor, and transforming growth factor-β 1 were reported to modulate the fracture healing process [8,9,10].

Parathyroid hormone-related protein (PTHrP), a family of protein hormones produced by most tissues in the body, was first discovered as the capital endocrine factor in the progress of humoral hypercalcemia of malignancy. However, PTHrP plays a crucial role in many normal physiological processes, namely, fetal bone development, cellular growth and differentiation, and mesenchymal–epithelial interactions [11,12]. To study the physiological function of PTHrP, Karaplis created PTHrP knock-out (KO) mice by a homologous recombination technology of embryonic stem cell [13]. PTHrP homozygote (PTHrP−/−) mice died of asphyxia shortly after birth, showing apparent dyschondroplasias. This clarifies that the most evident effect of PTHrP is to activate proliferation and inhibit differentiation and apoptosis of chondrocytes. However, the role of PTHrP changes in postnatal conditions, shifting its function to facilitate bone formation of osteoblasts. Mice that were selectively knocked out of PTHrP in osteoblasts displayed osteoporosis due to bone formation disorder. This study demonstrates the bone formation enhancement of endogenous PTHrP [14]. Skin-pop (SP) of PTHrP1-86 to mouse models of osteoporosis can not only activate 1,25-dihydroxyvitamin D(3) (1,25(OH)(2)D(3)) and promote bone formation but also save lives of the mice that have both PTH and 1α(OH)ase knocked out and regulate receptor activator of nuclear factor kappa B ligand and osteoprotegerin expression, which is to restrain bone resorption, and further prove the bone formation enhancement of exogenous PTHrP [15]. An in vitro study indicated that PTHrP could promote bone formation [16]. In another study, SP of synthetic human PTHrP1-36 to postmenopausal women showed that a certain dose of PTHrP could only promote bone formation selectively, and may decrease bone resorption. Hence, it may be an unalloyed bone formation accelerant superior to PTH [17].

According to previous studies, vertebra and hip fractures occur in 11.8% women and 13.8% men above 60 years of age [18]. The occurrence of high-energy fracture increases rapidly with the rise in traffic accidents. Since 10%–20% of fractures demonstrate healing complications and osteoporosis continues to be a debilitating disease, exploitation of bone-forming agents is of utmost importance. Endogenous and exogenous PTHrP have been shown to possess the ability to enhance bone formation. Previous studies demonstrated that haploinsufficiency of endogenous PTHrP impairs bone fracture healing [19] leading to speculation that exogenous PTHrP may be beneficial to bone fracture healing [20]. To confirm this supposition, we created and stabilized closed mid-diaphyseal femur fractures with intramedullary pins in eight-week-old wild-type (WT, PTHrP+/+) and PTHrP+/− mice in this study. The mice were then administered PTHrP (80 μg/kg) for one to four weeks post-fracture (PF) so as to explore the role of exogenous PTHrP in in vivo bone formation.

## 2. Results

### 2.1. Exogenous PTHrP Redressed Effect of Endogenous PTHrP-Deficient Delayed Bone Fracture Healing

**Figure 1:**
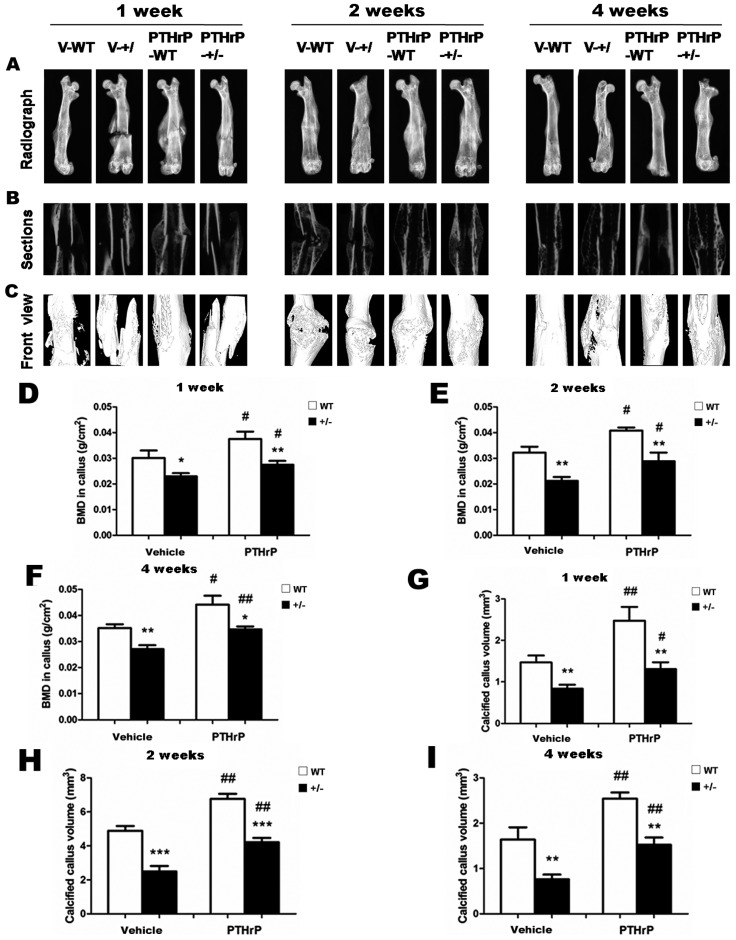
Exogenous parathyroid hormone-related protein (PTHrP) improves bone healing radiographic parameters in both wild-type (WT) and PTHrP-KO (knock-out) mice. Representative micrographs of callus sections and areas of the total callus from Vehicle (V)-WT, Vehicle-KO, PTHrP-WT, and PTHrP-KO mice at one, two, and four weeks post-fracture (PF) (**A**–**C**). Bone mineral density (BMD) (**D**–**F**) and calcified callus (**G**–**I**) were measured by computer-assisted image analysis.at one, two, and four weeks PF. Error bars are mean + SEM (standard error of mean). * *p* < 0.05; ** *p* < 0.01 and *** *p* < 0.001 compared with WT mice. # *p* < 0.05 and ## *p* < 0.01 compared with genotype-matched V-treated mice.

Effects of exogenous PTHrP on bone fracture healing were examined at one, two, and four weeks PF in vehicle wild-type (V-WT), V-KO, PTHrP-WT, and PTHrP-KO by X-ray (Figure 1A–C). The bone mineral density (BMD) increased after injecting PTHrP at one, two, and four weeks (Figure 1D–F). The size of callus and the calcified callus volume at one, two, and four weeks PF were less in PTHrP+/− mice compared with WT mice (Figure 1G–I). Nevertheless, the fracture repair process was promoted after administering exogenous PTHrP in PTHrP+/− mice and WT mice compared with vehicle-treated mice (Figure 1G–I).

### 2.2. Endogenous PTHrP Deficiency Inhibited Cartilage Differentiation and Exogenous PTHrP Promoted Cartilaginous Callus Formation and Transformation into Bony Callus

**Figure 2:**
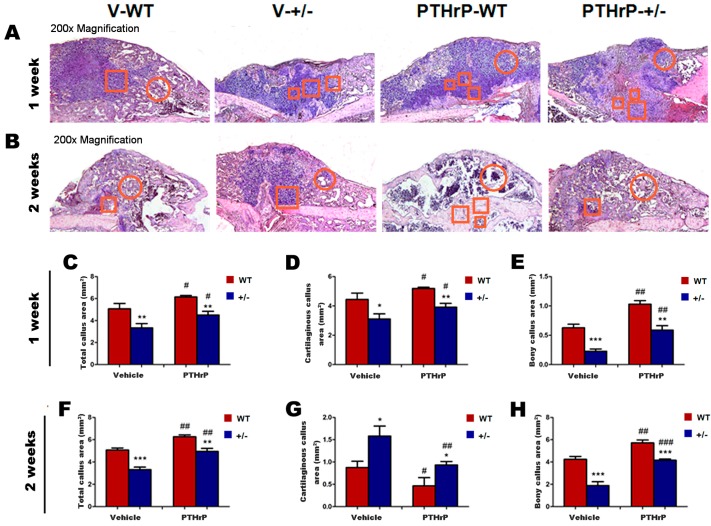
Effects of exogenous PTHrP on cartilaginous callus formation and on the transformation into bony callus. Representative micrographs of Trichrome staining in paraffin sections of calluses from Vehicle (V)-WT and V-PTHrP+/− mice, and PTHrP-WT and PTHrP+/− mice at (**A**) one week and (**B**) two weeks PF. Areas of the total callus (**C**,**F**), cartilaginous callus (**D**,**G**), and bony callus (**E**,**H**) were measured by computer-assisted image analysis. Error bars are mean + SEM. BMD: Bone mineral density; PF: post-fracture; PTHrP: parathyroid hormone–related protein; SEM: standard error of mean; WT: wild type. * *p* < 0.05; ** *p* < 0.01; and *** *p* < 0.001 compared with WT mice at the same group. # *p* < 0.05; ## *p* < 0.01, and ### *p* < 0.001 compared with genotype-matched vehicle-treated mice. Red circle area is bony callus and red square area is cartilaginous callus.

We analyzed callus tissues from WT and PTHrP+/− mice at one and two weeks PF using histology and computer-assisted image analysis to examine whether endogenous PTHrP deficiency affects cartilaginous callus formation and its transformation into bony callus. (Figure 2A,B). At one week PF, total callus areas, cartilaginous callus areas, and bony callus area decreased in PTHrP+/− mice compared with WT mice, but increased in PTHrP-treated WT and PTHrP+/− mice (Figure 2C–E). At two weeks PF, total callus areas and bony callus areas decreased in PTHrP+/− mice compared with WT mice, but increased in PTHrP-treated mice (Figure 2F,H). In contrast, at two weeks PF, remnant cartilaginous callus areas increased in PTHrP+/− mice compared with WT mice, but decreased in PTHrP-treated mice (Figure 2G).

### 2.3. Effects of Endogenous PTHrP Deficiency and Exogenous PTHrP on the Expression of Osteoblastic Bone Formation-Related Genes and Proteins in Callus Tissues

**Figure 3:**
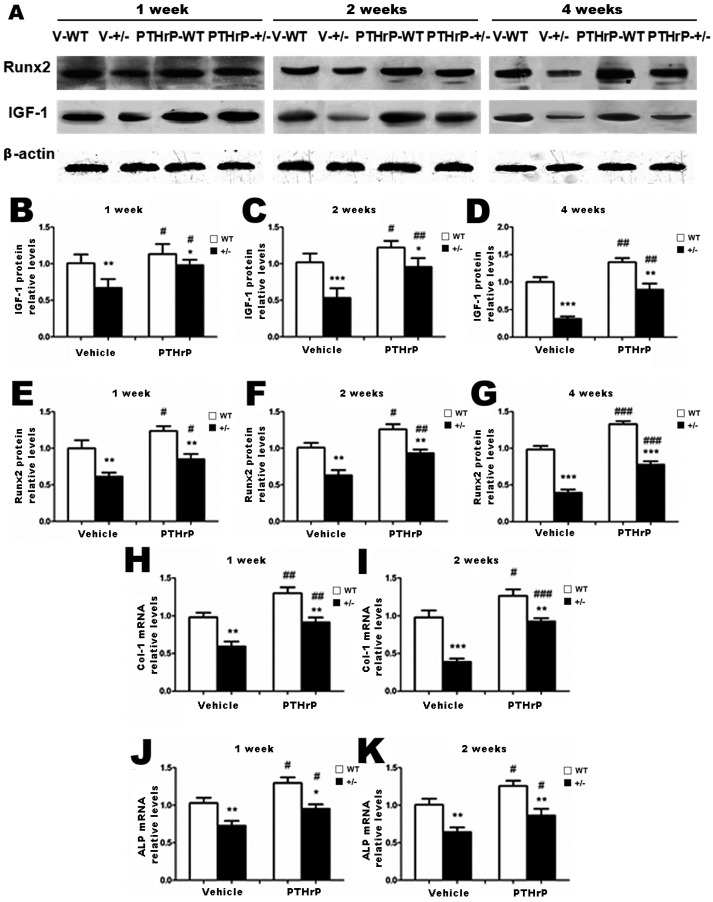
Exogenous PTHrP’s effects on the expression of osteoblastic bone formation–related genes and proteins in callus tissues at one, two, and four weeks PF (**A**). Expression of insulin-like growth factor-1 (IGF-1) and Runx-2 of callus extracts from Vehicle (V)-WT, V-KO mice, PTHrP-WT, and PTHrP-KO mice in western blots (**B**–**G**). Expression of alkaline phosphatase (ALP) and type I collagen (Col I) for callus extracts from V-WT, V-KO, PTHrP-WT, and PTHrP-KO mice in real time RT-PCR. (**H**–**K**). Error bars are mean + SEM. BMD: Bone mineral density; PF: post-fracture; KO: knock out; PTHrP: parathyroid hormone–related protein; RT-PCR: reverse transcriptase–polymerase chain reaction; SEM: standard error of mean; WT: wild type. * *p* < 0.05, ** *p* < 0.01 and *** *p* < 0.001 compared with WT mice. # *p* < 0.05, ## *p* < 0.01 and ### *p* < 0.001 compared with genotype-matched vehicle-treated mice.

We isolated proteins and mRNA from callus extracts of WT and PTHrP+/− mice and examined the association of the bony callus areas alteration with the regulation of osteoblastic bone formation-related genes and proteins’ expression [20]. We used Western blots and real time PCR (RT-PCR) to determine the expression of Runx-2 and insulin-like growth factor 1 (IGF-1) proteins, respectively, at one, two, and four weeks PF. We used RT-PCR to determine the mRNA levels of alkaline phosphatase (ALP) and type I collagen at one and two weeks PF. The protein levels of Runx-2 and IGF-1 at one, two, and four weeks PF (Figure 3B–G) and mRNA levels of ALP and type I collagen at one and two weeks PF (Figure 3H–K) decreased in PTHrP+/− mice compared with WT mice, but increased in PTHrP-treated mice (Figure 3B–K).

### 2.4. Effect of Endogenous PTHrP Deficiency and Exogenous PTHrP on Osteoblastic Bone Formation in Calluses

**Figure 4:**
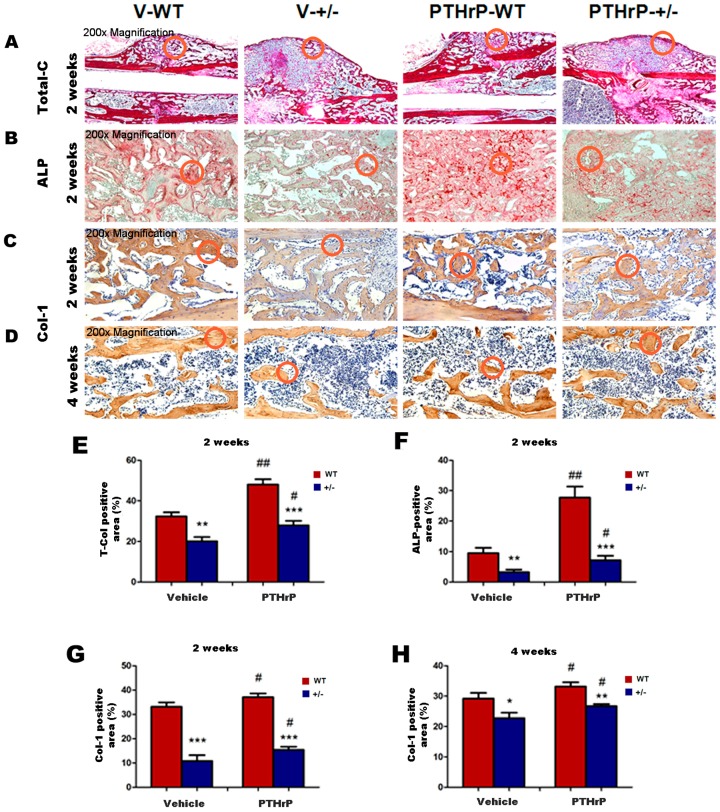
Effects of exogenous PTHrP on osteoblastic bone formation in calluses at two and four weeks PF. Representative micrographs of callus sections from Vehicle (V)-WT, V-KO, PTHrP-WT, and PTHrP-KO mice at two weeks PF stained with total collagen (**A**), histochemically for ALP (**B**), and immunohistochemically for type I collagen (Col-I) at two and four weeks PF (**C**,**D**). Total collagen-positive and ALP-positive bony callus areas at two weeks PF, and Col-I-immunopositive areas at two and four weeks PF (**E**–**H**). The positive areas are indicated using red circles. Error bars are mean + SEM. ALP: Alkaline phosphatase; BMD: bone mineral density; PF: post-fracture; KO: knock out; PTHrP: parathyroid hormone-related protein; SEM: standard error of mean; WT: wild type. * *p* < 0.05, ** *p* < 0.01 and *** *p* < 0.001 compared with WT mice. # *p* < 0.05 and ## *p* < 0.01 compared with genotype-matched vehicle-treated mice.

We measured bony callus volume and osteoblast number and activity at two and four weeks PF using histology, histochemistry, and immunohistochemistry to examine the effects of endogenous PTHrP efficiency and exogenous PTHrP on osteoblastic bone formation in calluses (Figure 4A–D). At two and four weeks PF, the total collagen-positive, ALP-positive, and type I collagen immunopositive bony callus areas all decreased in PTHrP+/− mice compared with WT mice, but increased in PTHrP-treated mice (Figure 4E–H).

### 2.5. Effects of Endogenous PTHrP Deficiency and Exogenous PTHrP on Osteoclastic Bone Resorption in Calluses at Two and Four Weeks PF

**Figure 5:**
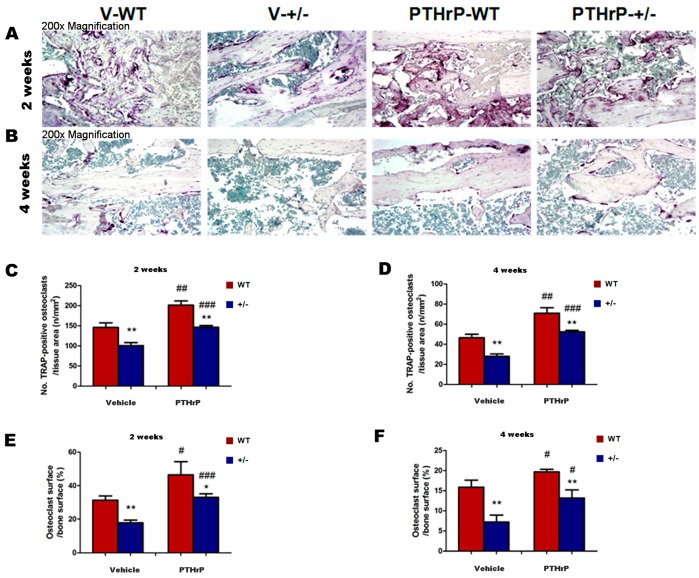
Exogenous PTHrP’s effects on osteoclastic bone resorption in calluses at two and four weeks PF. Representative micrographs of callus sections from Vehicle (V)-WT, V-KO, PTHrP-WT, and PTHrP-KO mice stained histochemically for tartrate-resistant acid phosphatase (TRAP) (**A**,**B**). Number of TRAP-positive osteoclasts related to tissue area (**C**,**D**) and osteoclast surface relative to bone surface (**E**,**F**), assessed by computer-assisted image analysis. Error bars are mean + SEM. KO: Knock out; PTHrP: parathyroid hormone–related protein; PF: post-fracture; SEM: standard error of mean; WT: wild type. * *p* < 0.05 and ** *p* < 0.01 compared with WT mice. # *p* < 0.05, ## *p* < 0.01 and ### *p* < 0.001 compared with genotype-matched vehicle-treated mice.

We measured osteoclastic bone resorption points in callus tissues at two and four weeks PF by histochemical staining and computer-assisted image analysis to examine whether endogenous PTHrP deficiency affect osteoclastic bone resorption during bone fracture healing (Figure 5A,B). The tartrate-resistant acid phosphatase (TRAP)-positive osteoclast number and surface decreased in PTHrP+/− mice compared with WT mice, but increased in PTHrP-treated mice at two and four weeks PF (Figure 5C–F).

### 2.6. Effects of Endogenous PTHrP Deficiency on Mechanical Properties of Femur Fracture Callus at Four Weeks PF

**Figure 6:**
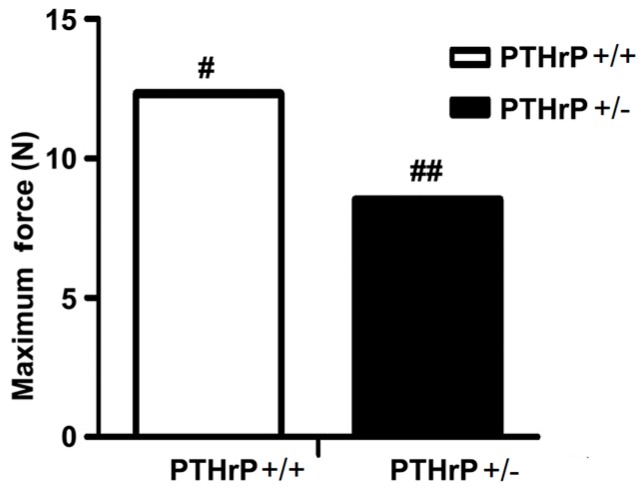
Effects of endogenous PTHrP deficiency on mechanical properties of fractured femurs at four weeks PF. Results revealed that maximum force at failure was less in PTHrP+/− mice compared with WT mice (*n* = 6). *# p* < 0.05 and *## p* < 0.01, WT mice compared with PTHrP+/− mice at the same group. PTHrP: Parathyroid hormone-related protein; WT: wild type.

A crucial property of bone healing is that the regenerated bone tissue must provide effective strength for the injured bone to regain its mechanical function [21]. To assess effects of endogenous PTHrP deficiency on mechanical properties of fractured femurs, biomechanical properties were examined by three-point bending in femurs four weeks PF in WT and PTHrP+/− mice. Results revealed that the maximum force at failure was less in PTHrP+/− mice (8.16 ± 0.82 N, *n* = 6) compared with WT mice (11.87 ± 1.27 N, *n* = 6). These data showed that endogenous PTHrP deficiency may reduce biomechanical properties in fractured femurs (Figure 6).

## 3. Discussion

Our study found that the size of callus and the calcified callus volume at one, two, and four weeks PF were less in PTHrP+/− mice compared with WT mice (Figure 1). Indeed, fractured femurs in PTHrP+/− mice recovered completely a few weeks later than WT, suggesting that endogenous PTHrP deficiency may result in delayed fracture healing. The fracture repair process was promoted in PTHrP+/− mice and WT mice after administering exogenous PTHrP compared with vehicle-treated mice. Meanwhile, at one week PF, total callus areas, cartilaginous callus areas, and bony callus area decreased in PTHrP+/− mice compared with WT mice, but increased in PTHrP-treated WT and PTHrP+/− mice (Figure 1 and Figure 2).

Our study confirms the previous finding that downregulation of osteoblastic genes and protein expression, and decreased endochondral bone formation, osteoblastic bone formation, and osteoclastic bone resorption are potential pathways through which the endogenous PTHrP deficiency impairs the bone fracture repair process by decreasing cartilaginous and bony callus formation [20]. Several studies have demonstrated that fracture healing is not a local process but rather a special wound repair type that involves complex cellular and molecular events, which includes thousands of different types of bioactive molecules from the fracture site [8,9,10]. Therefore, one of the major goals in modern fracture management is to enhance fracture healing, because it is of clinical importance in function recovery and regain after fracture.

Since fracture healing is likely to involve recruitment of osteoblast precursors differentiated from periosteum and bone marrow mesenchyme cell, it was reported that PTHrP may be involved in primary callus formation presumably cooperating with IGF-1 in osteoblasts and osteocytes, and by regulating chondrocyte differentiation in endochondral ossification [22]. As such, the present study hypothesized that bone fracture healing might be impaired in PTHrP mutant animal model.

Further, the results of this study demonstrated that at one and two weeks PF the mRNA levels of ALP, Runx-2, and type I collagen and the protein levels of Runx-2 and IGF-1 decreased in PTHrP+/− mice compared with WT mice (Figure 3), indicating that endogenous PTHrP plays an important role in bone fracture healing. These findings are in accordance with a previous study that reported that PTHrP-deficient mice display osteoporosis due to bone formation disorder [14,23]. Therefore, endogenous PTHrP’s critical role in endochondral bone formation during normal development may be duplicated in endochondral bone formation occurring during the fracture healing process [19]. Several studies have documented that exogenous PTHrP enhances bone formation in vivo and in vitro [15,16]. Moreover, another research revealed that PTHrP might induce osteoblastic activity and regulate fracture healing on the cortical bone surface [24]. Our findings are in accordance with the mechanism of endochondral bone formation course by revealing that at two and four week PF, the osteoblast number, ALP-positive areas, and type I collagen immunopositive areas all decreased in PTHrP +/− mice compared with WT mice, but increased in PTHrP-treated mice (Figure 4).

An experimental study demonstrated that mice with osteoblast-specific deletion of PTHrP exhibited impaired recruitment and increased apoptosis of osteogenic cells resulting in decreased bone formation and premature osteoporosis [25], thus PTHrP would serve as an early and effective identifier of individuals at risk of developing low bone mass and osteoporosis. The current study suggests that endogenous PTHrP affects bone BMD and shows that BMD increased after injecting PTHrP at one, two, and four weeks (Figure 1), therefore the PTHrP levels within the bone microenvironment might be critical in influencing bone mass acquisition [25]. Another study supported the conclusions of previous studies, suggesting that PTHrP functions as an anabolic agent to osteoblast [26].

A previous study reported that cell-derived PTHrP promotes osteoclastic bone resorption and contributes to the development and progression of cancer metastasis to bone [27]. The present study demonstrated that osteoclastic bone resorption or the TRAP-positive osteoclast number and surface decreased in endogenous PTHrP-deficient mice compared with WT mice (Figure 5). It also revealed that the maximum force at failure was less in PTHrP+/− mice (8.16 ± 0.82 N, *n* = 6) compared with WT mice (11.87 ± 1.27 N, *n* = 6), indicating that endogenous PTHrP deficiency may reduce biomechanical properties in fractured femurs (Figure 6). According to our data, fracture healing in PTHrP treated WT mice seems faster than in vehicle treated WT mice. However, whether the sharing force of PTHrP-WT fractured femurs is higher than that of WT shown was not examined in current study. We would like to investigate the sharing force of PTHrP-WT fractured femurs in the future. Our study confirms a previous finding that exogenous PTHrP promotes fracture repair by increasing callus formation and accelerating cell transformation [28], while endogenous PTHrP deficiency might result in osteoporosis and impaired bone formation in mice [20,29], which implies a potential application of exogenous PTHrP to repair bone damage and accelerate fracture healing clinically.

## 4. Materials and Methods

### 4.1. Sources of Mice

This study was reviewed and approved by the Institutional Review Board of Nanjing University Medical School, Nanjing, China (project No. NSFC81271997) in September 2012. Experiment animals were provided by the Bone and Stem Cell Laboratory of Nanjing Medical University, Nanjing, China. Animal care and treatment strictly followed the Guidelines on the Care and Use of Laboratory Animals of the Ethical Committee, Nanjing Medical University. All procedures performed were in accordance with the ethical standards of the institutions.

Eight-week-old littermate PTHrP+/− (PTHrP−/− mice died of asphyxia shortly after birth, thus PTHrP+/− mice were chosen) and wild-type (WT) mice were used in this study. The PTHrP+/− mice were a systemic KO of PTHrP, as confirmed via authors and by Miao and Wang’s methods [14,19,23].

### 4.2. Animal Fracture Models and Bone Tissue Preparation

The standard mice femur fracture models were generated from 30 eight-week-old WT mice and 30 eight-week-old PTHrP+/− mice, as reported in previous studies with minor modifications [19,20]. Six mice per group were used for histological sectioning and mechanical response tests. Briefly, after sedating the animals with isoflurane (Forene, Aerrane, Ohmeda, Liberty Corner, NJ, USA) and intraperitoneal anesthesia with a mixture of ketamine hydrochloride (80 mg/kg; Pfizer, New York, NY, USA) and 2% xylazine (12 mg/kg; Bayer, Leverkusen, Germany), we shaved and disinfected the right hind legs of the animals. A falling weight of 300 g with a height of 15 cm over a three-point bending mechanism was used to produce a mid-diaphyseal fracture. In all cases, the fracture was documented by radiography [20]. We introduced an intramedullary pin (Ø = 0.5 mm) into the femoral canal through a medial parapatellar incision and arthrotomy of the knee. Previous data show that a fracture generated in this manner heals through both endochondral and intramembranous ossification [30]. At the time of fracture, WT and PTHrP+/− mice received daily injections of saline or of PTHrP (80 µg/kg, Biovision, Milpitas, CA, USA) subcutaneously for two weeks.

A set of six mice were killed by anesthesia at scheduled intervals for each fracture healing time point (one, two, and four weeks PF). The fracture calluses were harvested and processed, three for RNA extraction and three for Western blotting. For RNA and protein extraction, the harvested tissues were snap-frozen in liquid nitrogen and immediately stored at −80 °C until processing. For a set of three mice, fracture femur shafts were prepared and immediately fixed in 4% paraformaldehyde overnight at 4 °C, followed by a standard protocol of dehydration and paraffin embedding.

### 4.3. Skeletal Radiography

The fracture models were affirmed by X-ray after operation at one, two, and four weeks PF. If the fracture produced was not stable or if deep infection developed, the animal was excluded from the study and replaced with another animal. Radiographs of the fractured legs were serially taken using a Faxitron model 805 radiographic inspection system (Faxitron Contact, Faxitron, Much, Germany) (22 kV voltage and 4-min exposure time) at one, two, and four weeks PF after the fracture. X-Omat TL film (Eastman Kodak Co., Rochester, NY, USA) was used and processed routinely. This procedure was performed by removing limbs under anesthesia quickly. Then the limb calluses were harvested immediately and snap-frozen in liquid nitrogen and immediately stored at −80 °C for RNA and protein study. Fracture union was identified by the presence of bridging callus on two cortices.

### 4.4. Micro Computed Tomography

We analyzed femurs using microcomputed tomography (micro-CT) and associated analysis software (SkyScan 1072 scanner, Antwerp, Belgium), as described in previous studies [20,26]. In brief, we enclosed samples in tightly fitting plastic wrap to prevent movement and dehydration, and obtained images at 100 kV and 98 mA with a 0.98 rotation between frames. To segment the bone from the background, we used thresholding on the images. The resolution of the micro-CT images was 9.1 μm. The instrument provided a 3D Creator software to generate three-dimensional (3D) renderings from 2D images.

### 4.5. RNA Preparation and Quantitative Real-Time Reverse Transcriptase–Polymerase Chain Reaction

RNA was isolated from callus tissues using Trizol reagent (Invitrogen, Carlsbad, CA, USA), as described in a previous study [20]. Reverse transcription reactions were performed by adopting the SuperScript First-Strand Synthesis System (Invitrogen), as described in a previous study [26]. Messenger RNA expression was calculated as a ratio relative to glyceraldehyde-3-phosphate dehydrogenase (GAPDH) mRNA levels and expressed relative to V-WT group levels. In brief, real time polymerase chain reaction (RT-PCR) analyses were performed using the LightCycler System (Roche, Indianapolis, IN, USA) to determine the number of cDNA molecules in the reverse transcribed samples. PCR was performed using 2-mL LightCycler DNA Master SYBR Green I (Roche), 0.25 mM of 5′ and 3′ primers, and 2-mL samples or H_2_O to a final volume of 20 mL. The concentration of MgCl_2_ was 3 mM. Sample denaturation, amplification, and fluorescence determination were carried out as described in a previous study [20]. Purified PCR fragments of known concentration were serially diluted and served as external standards for each experiment to determine the number of copies of the targeted DNA in the samples. GAPDH levels in the samples were used to normalize the data. The primer sequence used for the real-time PCR was the same as that described in previous studies (Table 1) [20,31].

### 4.6. Western Blot Analysis

Proteins were extracted from six callus tissues of each group and quantitated using a kit (Bio-Rad, Mississauga, ON, Canada). Protein samples (30 μg) were fractionated by sodium dodecyl sulfate polyacrylamide gel electrophoresis and transferred to nitrocellulose membranes. Immunoblotting was carried out as described in a previous study [32] using antibodies against insulin-like growth factor 1 (IGF-1) (Santa Cruz, CA, USA) and Runt-related transcription factor 2 (*Runx-2*) (Santa Cruz, CA, USA) with β-tubulin (Santa Cruz, CA, USA) used as loading control. Runx-2 and IGF-1 protein levels relative to β-actin protein amounts were assessed by densitometric analysis and expressed relative to the levels obtained for V-WT mice [33]. Bands were visualized using enhanced chemiluminescence (Amersham, Aylesbury, UK).

### 4.7. Histology

Femurs were removed from six mice of each group and fixed in periodate–lysine–paraformaldehyde fixative (2% paraformaldehyde containing 0.075 M lysine and 0.01 M sodium periodate) overnight at 4 °C and processed histologically, as described in a previous study [34]. Femurs were decalcified in ethylenediaminetetraacetic acid (EDTA)–glycerol solution for five to seven days at 4 °C. Decalcified femurs were dehydrated and embedded in paraffin, then 5-mm sections were cut on a rotary microtome. The sections were stained with hematoxylin and eosin (HE) or histochemically for total collagen and alkaline phosphatase (ALP) activity and tartrate-resistant acid phosphatase (TRAP) or immunohistochemically, as described in a previous study [20].

### 4.8. Immunohistochemical Staining

The avidin–biotin–peroxidase complex technique with affinity-purified goat anti-human type I collagen antibody (Southern Biotechnology Associates, Birmingham, AL, USA) was used to perform immunohistochemical staining for type I collagen on paraffin sections [20,35]. In brief, we incubated dewaxed and rehydrated paraffin-embedded sections with methanol–hydrogen peroxide (1:10) to block endogenous peroxidase activity and then washed them in Tris-buffered saline (pH 7.6). The slides were then incubated with the primary antibody at room temperature overnight. The tissues were rinsed with Tris-buffered saline for 15 min, then incubated with biotinylated secondary antibody (Sigma-Aldrich, Stockholm, Sweden). Finally, the sections were counterstained with Mayer’s hematoxylin, dehydrated in graded ethanol and xylene, and mounted with Biomount medium, after washing with distilled water [20].

### 4.9. Biomechanical Testing

Fractured femurs were collected four weeks after the fracture. Femurs of six WT and PTHrP+/− mice were carefully dissected free of surrounding soft tissues, and placed in phosphate-buffered saline at room temperature. We used a three-point bending test to assess their biomechanical properties [36]. Right femur midshaft (span length, 10 mm) was used to perform strength tests using a machine (Instron, Norwood, MA, USA) with a displacement rate of 10 mm/min. Load-deflection diagrams were used to measure whole-bone mechanical properties, including maximum load, maximum stress, and energy to failure. Maximum stress was calculated as *s* = *yFL*/4*I*, where *s* = maximum stress (N/mm^2^), *y* = perpendicular distance from neutral axis (mm), *F* = load (N), *L* = length between two supports (mm), and *I* = moment of inertia (mm^4^).

### 4.10. Computer-Assisted Image Analysis

Images of fields were photographed after HE staining or histochemical or immunohistochemical staining of sections from six mice of each genotype. Images from single sections were digitally recorded using a rectangular template and analyzed using Northern Eclipse image analysis software (Empix Imaging, Inc., Mississauga, ON, Canada), as described in previous studies [23]. We defined the region of interest (ROI) as the area of the callus on both sides of the medullary canal excluding all cortical bone. This ROI was outlined manually for each specimen by using the software, as we have described elsewhere [19].

### 4.11. Statistical Analysis

Data were shown as means ± standard error of means (SEM) of at least three independent experiments. The data were analyzed by one-way analysis of variance with Tukey’s multiple comparison post hoc test. Statistical analyses were performed using the PRISM version 6.0 software (Graph-Pad Software, La Jolla, CA, USA). A *p* < 0.05 was considered to be statistically significant.

## 5. Conclusions

In conclusion, the results of the current study showed that endogenous PTHrP deficiency may result in delayed fracture healing, and demonstrated the important role played by exogenous PTHrP in fracture healing. Exogenous PTHrP affects osteoblast and osteoclast by increasing callus areas, endochondral bone formation, and callus remodeling, and produces a bone of greater mechanical strength. Further research is necessary to explain the relative importance of PTHrP genetics.

## Figures and Tables

**Table 1:** Primer sequence used for PCR.

Gene	Nucleotide Sequence
*ALP*	sense: 5′–3′CTTGCTGGTGGAAGGAGGCAGG
	anti-sense: 5′–3′GGAGCACAGGAAGTTGGGAC
*Runx*-2	sense: 5′–3′CTTCATTCGCCTCACAAACA
	anti-sense: 5′–3′TTGATGCCATAGTCCCTCCT
*Col*-I	sense: 5′–3′TCTCCACTCTTCTAGTTCCT
	anti-sense: 5′–3′TTGGGTCATTTCCACATGC
*GAPDH*	sense: 5′–3′GGTCGGTGTGAACGGATTTG
	anti-sense: 5′–3′ATGAGCCCTTCCACAATG

ALP: Alkaline phosphatase; Col-I: type I collagen; GAPDH: glyceraldehyde-3-phosphate dehydrogenase; PTHrP: parathyroid hormone–related protein; Runx-2: Runt-related transcription factor 2.
